# Perceptions of Workplace Heat Exposure and Controls among Occupational Hygienists and Relevant Specialists in Australia

**DOI:** 10.1371/journal.pone.0135040

**Published:** 2015-08-19

**Authors:** Jianjun Xiang, Alana Hansen, Dino Pisaniello, Peng Bi

**Affiliations:** 1 School of Public Health, The University of Adelaide, Adelaide, South Australia, Australia; 2 Department of Public Health Preparedness and Response, Fujian Provincial Centre for Disease Control and Prevention, Fuzhou, Fujian Province, China; The Ohio State University, UNITED STATES

## Abstract

With warmer weather projections, workplace heat exposure is presenting a growing challenge to workers’ health and safety. Occupational hygienists are the specialist group conducting measurements and providing advice on heat stress management to industry. In order to provide insights into hygienists perceptions on workplace heat exposure, current and future preparedness for extreme heat, and barriers to possible heat adaptation strategies, a self-administered questionnaire survey was conducted during a national conference of the Australian Institute of Occupational Hygienists. Nearly 90% of the 180 respondents were at least moderately concerned about extreme heat and 19% were dissatisfied with current heat stress prevention measures. Barriers recognized by the participants were lack of awareness (68%), insufficient training (56%), unsatisfactory management commitment (52%), and low compliance with prevention policies (40%). The findings suggest a need to refine occupational heat management and prevention strategies.

## Introduction

The potential for an increase in heat stress due to climate change in occupational settings and its potential impacts on productivity loss have been addressed in recent years [1–4]. Australian average temperatures are projected to rise by 1.0 to 5.0°C by 2070 and the number of heatwaves are expected to triple by 2050 [5], when compared with the climate of recent decades. Evidence has shown that higher average temperatures and increasing periods of extremely hot weather may place outdoor physical workers in Australia at moderate to high risk of heat strain by 2070 [6]. This may result in a significant reduction in afternoon work time [7, 8], as well as possible increases in heat-related illnesses and injuries [2, 4, 9, 10].

Heat illnesses are largely preventable. Heat risk awareness and knowledge are an important integral part of heat preventive measures. Effective heat stress management in the workplace requires comprehensive efforts and cooperation from a range of stakeholders including government organizations, occupational health and safety (OH&S) service providers, employers, unions and workers. Better understanding of how people involved in OH&S at different levels view the risk of workplace heat exposure will help to inform heat prevention and adaptation strategies and practical guidelines in a warming climate.

Occupational health and safety specialists (e.g. industrial hygienists) are at the frontline protecting workers’ health and safety by recognizing, evaluating, and controlling environmental hazards. As professionals, they play central and crucial roles in heat stress training, monitoring, assessment and management, and prescribe heat prevention recommendations to employers. Hence, their perspectives are of particular importance and may be helpful when considering heat-related health and safety reforms in the context of climate change. This study aims to provide insights into the Australian occupational hygienists’ and related specialists’ perceptions on this issue and their views on current and future preparedness for extreme heat.

## Materials and Methods

A cross-sectional self-administered survey was conducted during the 30^th^ Annual Conference & Exhibition of the Australian Institute of Occupational Hygienists Inc. (AIOH) held in Adelaide from 1^st^ to 5^th^ December 2012. The study was approved by both the Human Research Ethics Committee at the University of Adelaide (H-200-2011, S1 Appendix) and the conference organizing committee.

### Questionnaire design

The design of the survey questions was guided by both relevant literature [2, 11–13] and our research aims and objectives. The instrument (S2 Appendix) contained three demographic questions (position, years of OH&S experience, and State/Territory) and questions focusing on the following aspects. First, to measure concern and awareness of current workplace extreme heat exposure and climate change, three 5-point Likert-scale questions were asked. Second, to investigate participants’ heat stress management experience and companies’ preparedness for a warming climate, eight specific items were developed. Third, to understand current heat preventive measures and identify existing adaptation barriers, participants were asked: (1) “What measures are adopted in the workplace or workplaces that you consult in during very hot weather?” and (2) “What do you foresee as potential barriers for the prevention of heat stress in the workplace?” The final question was open-ended: “Do you have any further recommendations or suggestions for the prevention of heat-related illnesses and injuries in Australian workplaces?” Additionally, some secondary questions had open-ended responses.

The survey instrument was pre-tested for length, clarity and comprehension with a convenience sample of five occupational hygienists from The University of Adelaide. Minor modifications were made according to the feedback before the instrument was finalized.

### Participant recruitment

A purposive convenience sampling method was used [14]. With the support of the AIOH conference organizing committee, 371 registered conference delegates (professional hygienists and relevant occupational specialists) in attendance were invited to voluntarily participate in the study. Questionnaires (S2 Appendix) and information sheets (S3 Appendix) were distributed to the delegates at the conference registration desk where completed questionnaires were placed in a return box. Oral consent was obtained from each participant. The chance to win a donation to charity and a Christmas gift were offered as an incentive for participation.

### Data analyses

Data were entered into Microsoft Excel 2010. Data analyses were conducted using Stata v12.0 (Stata Corp LP, College Station, Texas). Descriptive analysis was used to calculate the frequency of variables. Chi-square tests were used to investigate the correlation between heat concern, satisfaction, and attitudes towards further heat prevention efforts, and to compare the differences in heat concern between occupational hygienists and other specialists in the area. In addition, due to the booming mining industry (where heat stress can be common) in Western Australia and Queensland in recent years [15], the heat concern level between participants from these States and other Australian States was compared.

## Results

As shown in Table 1, 180 out of the 371 conference delegates participated in the survey during the 5-day conference period, with a participation rate of 48.5%. Among the 180 participants, 170 (94.4%) had OH&S work experience, ranging from 1 to 44 years, with an average of 16 years. There were 89 occupational hygienists, representing about half (49.4%) of all respondents, followed by occupational consultants (26.7%), staff from companies providing OH&S services (21.1%), and workplace OH&S managers (2.8%). Thirty-five (39.3%) out of the 89 occupational hygienists were general industrial hygienists. Others were from areas including mining, government and university sectors. The majority of participants were from Western Australia, followed by New South Wales, Victoria, South Australia, and Queensland.

**Table 1:** Demographic information of the participants.

Category	n	%
**Total**	180	100.0
**Occupation**		
Hygienist	89	49.4
Occupational consultant	48	26.7
OH&S manager	5	2.8
Other	38	21.1
**Area**		
Western Australia	45	25.0
New South Wales	37	20.6
Victoria	30	16.7
South Australia	22	12.2
Queensland	18	10.0
Overseas	13	7.2
Tasmania	9	5.0
Australian Central Territory	4	2.2
Northern Territory	2	1.1

### Concern and awareness

As shown in Table 2, nearly 90% of participants were moderately or more concerned about extreme heat resulting in increased hazards in the workplace. Almost two thirds of participants agreed that extremely hot weather due to a changing climate may present a future challenge for heat stress management. Half of participants were satisfied or strongly satisfied with the heat preventive measures currently adopted in workplaces. By contrast, 19.4% were not satisfied.

**Table 2:** Number and percentage of respondents' concern and awareness on workplace extreme heat exposure.

Question	Response	n	%
(1) How concerned are you about extreme heat resulting in increased hazards in the workplace?	Extremely	27	15.0
	Very	93	51.7
	Moderately	43	23.9
	A little	13	7.2
	Not at all	4	2.2
(2) Do you agree or disagree that extremely hot weather due to changing climate presents a future challenge for workplace heat management?	Strongly agree	21	11.7
	Agree	96	53.3
	Neutral	44	24.4
	Disagree	15	8.3
	Strongly disagree	4	2.2
(3) Overall, are you satisfied or dissatisfied with the measures currently adopted for reducing the risk of heat illnesses and injuries in the workplace or workplaces that you consult in during very hot weather?	Strongly satisfied	5	2.8
	Satisfied	85	47.2
	Neutral	55	30.6
	Dissatisfied	35	19.4
	Strongly dissatisfied	0	0.0

As shown in Table 3, no significant differences were observed between occupational hygienists and others on heat concern and attitudes toward likely increasing heat stress challenge due to climate change, and satisfaction level of current heat prevention measures. Similarly, no significant differences were identified between Western Australia and Queensland and other Australian States.

**Table 3:** Comparison of heat concern, attitudes toward climate change related heat challenge, and satisfaction level of heat prevention measures by occupation and area (Chi-square test).

	Heat concern			Future heat stress challenge			Satisfaction of heat prevention measures		
	Yes	No	p-value	Yes	No	p-value	Yes	No	p-value
**Occupation**									
Hygienists	63	26	0.246	58	31	0.963	48	42	0.297
Non-Hygienists	57	34		59	32		41	49	
**Area**									
WA and Qld*	43	20	0.740	36	27	0.105	36	27	0.130
Other States	77	40		81	36		53	64	

*WA and Qld: Western Australia and Queensland.

### Preparedness and management

As shown in Table 4, 68.9% of participants indicated that there were hot weather plans or heat stress policies currently available in their workplaces or workplaces where they consult. When further asked which industrial sectors had hot weather plans or polices (data not shown), 62.1% mentioned the mining industry, followed by manufacturing (25.0%), electricity, gas and water (8.9%), construction (8.1%), and defence (3.2%). Ninety percent of participants responded that they had heard workers express concerns over heat during hot weather, of which 7.4%, 36.8% and 44.2% said “always”, “often”, and “sometimes”, respectively. About half (52.8%) of respondents reported that they have investigated heat-related illnesses or injuries in the past five years.

**Table 4:** Number and percentage of extreme heat preparedness and management.

Question	Number (%)		
	Yes	No	Not sure
(1) Is there a hot weather plan or heat stress policy in your workplace or any workplaces that you consult in?	124 (68.9)	42 (23.3)	14 (7.8)
(2) In your experience have workers ever expressed concern about heat in your workplace (or workplaces you consult in) during very hot weather?	162 (90.0)	18 (10.0)	-
(3) In the last five years, have you had to investigate the circumstances around injuries or illnesses that could be attributed to extreme heat (air temp greater than 38°C)?	95 (52.8)	85 (47.2)	-
(4) Do you think there is a need for more training about working in very hot weather in your workplace or workplaces that you consult in?	109 (60.6)	69 (38.3)	2 (1.1)
(5) Do you think there should be more regulations for reducing the risk of heat-related illnesses and injuries in very hot weather applicable to your workplace or workplaces that you consult in?	93 (51.7)	66 (36.7)	21 (11.7)
(6) Do you know of any organizations planning for increased frequency of extremely hot weather events?	33 (18.3)	147 (81.7)	-
(7) Do you know of any companies that have recently made changes to maintain OH&S in work environments where extreme heat may become more common?	48 (26.7)	132 (73.3)	-
(8) Do you intend to alter your recommendations to management or companies due to the likelihood of increased hot weather?	47 (26.1)	95 (52.8)	38 (21.1)

About 61% of respondents agreed that there was a need to increase heat-related training in workplaces. Approximately one third held the opposite view, with 85.3% believing that “training is generally adequate” and 14.8% considered “it is not a serious problem” (data not shown). When asked which aspects of heat-related training should be strengthened, respondents suggested:

*“Hydration maintenance*, *self-pacing*, *heat acclimatization*, *and early symptoms of heat illness”*, *“impact of personal protective equipment on human body heat balance maintenance”*, *“individual heat risk factors e*.*g*. *predisposing medical conditions*, *lifestyle*, *fitness level”*, *“annual training prior to hot seasons”*

When asked if there is a need for more heat-related regulations, more than half answered “yes”, 36.7% said “no”, and 11.7% replied “not sure”. Among those with negative responses, most thought “there are enough heat-related regulations already” and 11.1% considered “it is not a serious problem” (data not shown).

The results showed that most (81.7%) respondents did not know of organizations planning for the predicted increase of extremely hot weather. Furthermore, 73.3% of respondents did not know of companies that have made changes to reduce the impact of extremely hot weather. Only 26.1% of respondents intended to alter their heat-related prevention recommendations. By contrast, 52.8% of respondents did not want to change their recommendations to management or companies.

### Preventive measures and adaptation barriers

**Fig 1:**
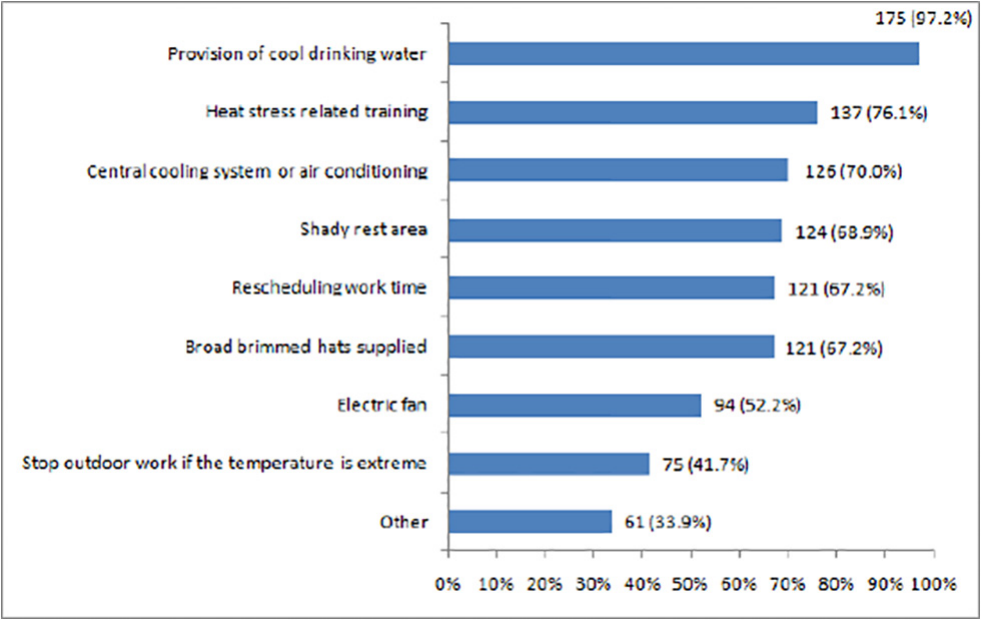
Heat-related preventive measures currently adopted in the workplace.

As shown in Fig 1, “provision of cool drinking water” was the most commonly mentioned preventive measure currently adopted in the Australian workplaces for reducing the impact of heat exposure, followed by “heat stress related training” (76.1%), “central cooling system or air conditioning” (70.0%), “shady rest area” (68.9%), “rescheduling work time” (67.2%), and “electric fan” (52.2%). Furthermore, 41.7% of participants recommended “the cessation of work if the temperature is extreme”. Other heat prevention measures suggested by the respondents in open-ended responses included:

*“Standardized heat checklist”*, *“personal heat stress monitoring”*, *“heat vulnerability screening”*, *“more training and research”*, and *“raising awareness through school education and mass media”*.

**Fig 2:**
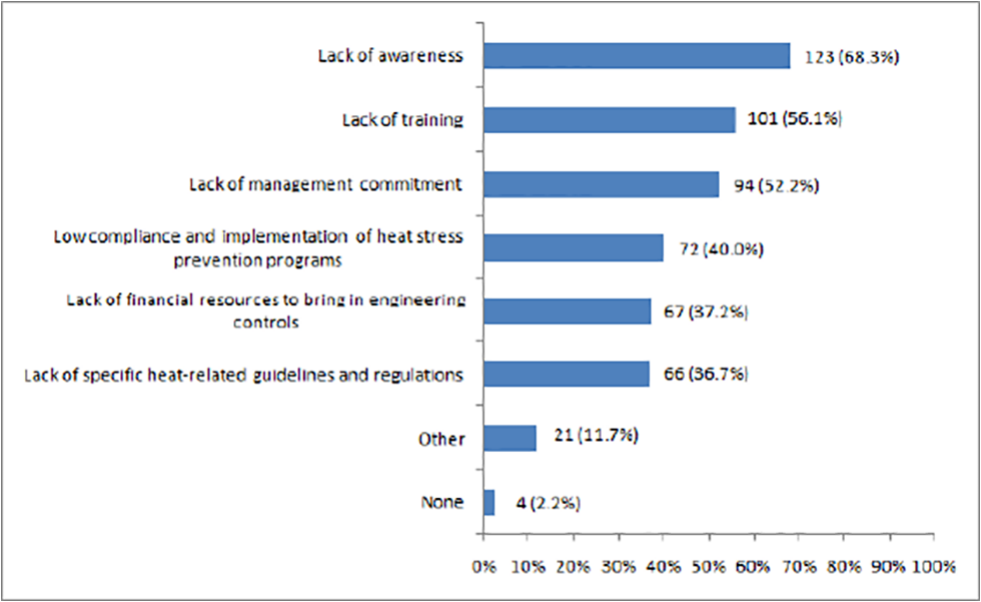
Heat prevention and adaptation barriers existing in the workplace.

As shown in Fig 2, the most frequently mentioned heat prevention and adaptation barrier was “lack of awareness” (68.3%), followed by “lack of training” (56.1%), “lack of management commitment” (52.2%), “low compliance and implementation of heat stress prevention programs” (40.0%), “lack of financial resources to bring in engineering controls” (37.2%), and “lack of specific heat-related guidelines and regulations” (36.7%). Other potential barriers suggested by the respondents were:

*“The conflict between increased personal protective equipment requirements and heat dissipation”, “work demands/priority”, “peer pressure”, “workers’ poor self-management”, “lack of engineering controls”, “lack of compliance”, “lack of hydration awareness”, “cultural and ethnic issues”, “difficulties in implementing controls in mobile workplaces”,* and *“lack of heat acclimatization due to the fly-in/fly-out (FIFO) [16] work regime”.*

### Relationship between heat concern, satisfaction and heat prevention efforts

As shown in Table 5, participants who have concerns about heat exposure were more likely to support the strengthening of heat-related training and regulations, and also more likely to alter their recommendations to their management team or companies. Those not satisfied with current heat prevention measures showed stronger views on more heat-related training and regulations. However, no differences were detected in the adjustment of heat prevention advice between those satisfied or not satisfied with current heat prevention measures.

**Table 5:** Relationship of heat concern and satisfaction level on the attitudes towards more training and regulations, and the adjustment of heat prevention recommendations (Chi-square test).

Classification	More training required			More regulations required			Suggested amending recommendations		
	Yes	No	p-value	Yes	No	p-value	Yes	No	p-value
**Concern about heat exposure**									
Yes	82	38	0.003	75	45	<0.001	40	80	0.002
No	27	33		18	42		7	53	
**Satisfied with heat prevention measures**									
Yes	45	45	0.004	40	52	0.025	21	69	0.396
No	64	26		53	35		26	64	

## Discussion

Perceptions about weather-related heat stress and its potential impacts on OH&S have been investigated among South African outdoor labourers [11], Indian manufacturing workers [13], US migrant farmers [17], and Australian workplace OH&S representatives [18]. However, to date there is no research investigating the perceptions and activities from the perspective of professionals such as occupational hygienists who work at the coalface in controlling environmental and occupational hazards. Therefore, this study, the first of its kind in Australia, using a quantitative questionnaire survey, provides valuable baseline information and expert opinion on heat-related OH&S hazards. The results will provide implications for the development of heat stress management and policies.

### Heat exposure concern

The results of this survey showed that a vast majority of occupational hygienists and specialists were concerned about workplace heat exposure. Almost all respondents often heard workers’ concerns on heat exposure during hot weather, indicating that workers’ thermal comfort has been compromised by heat exposure in Australian workplaces. This is supported by previously published studies investigating workers’ physiological responses to heat exposure [19–21]. For example, a study conducted in Western Australia showed that nearly 70% of aboveground miners were poorly hydrated [19]. Heat strain has also been reported among South Australian shearers, carpenters, and railway track maintenance workers when temperatures exceeded 37 C [20]. Moreover, in the present study more than half of respondents had the experience of investigating injuries and accidents occurring during extremely hot weather, supporting the notion that the occurrence of serious heat-related illnesses, injuries and even deaths in Australian workplaces is not uncommon [22–25]. This suggests that heat stress prevention methods currently adopted in Australian workplaces may not sufficiently and effectively protect vulnerable workers from heat-related illnesses and injuries. Relevant actions should be taken on the aspects of administrative controls, educational training, personal behaviours adjustment, and the development of heat prevention policies.

With projections of increasing temperatures due to climate change [5], heat exposure may present a growing challenge to workers’ health and safety [2, 4, 6]. The results in this study showed that a majority of occupational hygienists and specialists agreed with this view. However, only 26% of participants intended to amend their heat-related prevention recommendations to companies. The awareness-practice gaps may be explained by the fact that some participants believed that climate change related heat impacts on OH&S are not a current threat. Using a respondent’s words from an open-ended question, it is “*not confirmed science*”. The two major reasons accounting for some occupational specialists’ lack of concern over heat exposure were: (1) they felt there were adequate measures already being implemented; and (2) scepticism about the realistic impact of climate change on workers’ health and safety. As occupational hygienists and specialists play crucial roles in heat stress controls, therefore specific educational programmes (e.g. heat-related training, workshops, or forums) may be required to strengthen their awareness of the impact of climate change on workers’ health and safety.

### Heat exposure preparedness

From the surveyed delegates’ point of view in this survey, Australian workplaces are not well-prepared for the likelihood of a warmer climate, as most of the skilled OH&S professionals did not know of any companies that have taken actions to reduce the impact of heat stress. This is consistent with the findings of a qualitative research study in Australia which found that: “current heat exposures are already at a hazardous level and existing workplaces are ill-equipped to protect workers’ health” [18]. Although most workplaces reportedly had hot weather plans, previous studies have shown a significant increase of injury claims for vulnerable sub-groups during heatwaves in Adelaide [26].

When asked which industries had a hot weather plan, the respondents mentioned mining, manufacturing, ‘electricity, gas and water’, construction, and defence. Evidence has also shown that farmworkers and forestry workers are at high risk of heat illnesses and injuries [10, 27–31], especially seasonal and migrant workers [32–34]. However, occupational hygienists and relevant specialists are not usually employed in these sectors. Australia has a large group of seasonal and migrant farmworkers due to farm labour shortages, relatively high hourly wages, and the provision of working holiday visas (sub-class 416 and 417) [35]. Agricultural workers, especially migrant and seasonal farm labourers, are one of the most underserved and understudied occupational populations [33]. Therefore, more heat prevention programmes should be directed to this heat vulnerable group.

### Current heat prevention measures

The results showed that about half of respondents were not satisfied with current heat prevention measures, reflecting the necessity to upgrade current heat policies and strategies. Australia has mandatory heat regulations in place to protect workers from extreme heat; however, at present there are many ambiguities which may hinder implementation and effectiveness [4, 36]. According to the survey results, provision of cool drinking water was the most common heat prevention measure, which is also required by the national Model Code of Practice (managing the work environment and facilities) [37]; however, this is not available in all Australian workplaces according to the results of this study. Some non-governmental institutions (e.g. the Australian Institute of Occupational Hygienists Inc. (AIOH) [38] or trade unions (e.g. the Construction, Forestry, Mining and Energy Union (CFMEU) [39]) have made specific heat stress management guidelines. Nevertheless, the unenforceable and non-mandatory nature of the guidelines may raise the problem of low compliance [36, 40].

According to the Australian Construction, Forestry, Mining and Energy Union (CFMEU), “if temperature is over 37°C all work ceases unless working in air conditioned area” [39]. However, in this study only 40% of respondents selected “stop outdoor work” as a preventive measure when the temperature was extremely hot. Some workers such as emergency (e.g. fire-fighting) and utility maintenance staff are inevitably exposed to extreme heat due to the nature of tasks. In this case, the incorporation of alternative technologies and real time health surveillance may be a solution. For example, more breathable personal protective equipment could be developed or the wearing of cooling vests considered.

### Heat prevention barriers

In this study, a majority of Australian occupational hygienists and specialists thought that lack of awareness was the major barrier for heat stress prevention. This is supported by a study conducted in the USA which had similar findings [17]. Therefore, in 2011, the US OSHA launched a nationwide campaign to raise heat risk awareness in the workplace [41]. Heat prevention barriers may also include lack of training and management commitment, low compliance and implementation of heat prevention policies, and lack of financial resources, which have been identified by previous heat studies in the USA [36, 42]. In addition, the increase of the fly-in/fly-out (FIFO) work regime may prove a challenge for heat acclimatisation [15]. Currently, approximately 49% of Western Australia’s mining sites are operating on a FIFO basis [15]. If workers are flown in from a cool area to an extremely hot area, they may be not acclimatized to heat and would be more susceptible to heat-related injuries.

### Limitations

The limitations to this study include that a purposive convenience sampling method was employed. The sampling framework is restricted to delegates of a conference, which may generate potential selection bias. Due to the relatively small sample size, caution should be exercised regarding the generalization of the results. The views cannot be assumed to represent those of all occupational hygienists in Australia. Nevertheless, a large proportion of senior occupational hygienists in Australia participated in the survey according to the consultation with AIOH. Therefore, results of this study may basically represent the majority of Australian occupational hygienists’ and specialists’ opinions on workplace heat exposure. Further research is needed to: (1) explore why a considerable proportion of occupational hygienists and associated professionals were not satisfied with current heat prevention measures, (2) investigate how workers, employers, policy-makers and relevant stakeholders perceive the risk of workplace heat exposure, and (3) measure current heat stress level in some heat vulnerable occupations and identify trends in temperature change to provide more solid evidence about workplace heat exposure. In addition, more in-depth qualitative research is needed to further explore heat risk perceptions, although results of open-ended questions in this study provide a supplement to this survey. Despite the limitations, this survey may add to current knowledge and provide valuable expert opinion in terms of the development of heat prevention strategies.

## Conclusions

Australian occupational hygienists and specialists showed concerns over heat stress, but did not show strong willingness to amend heat prevention recommendations to management or companies. The Australian workplaces may not be well-prepared for the likelihood of increasing incidence of heat stress due to climate change. The major heat prevention barriers recognized by the participants were lack of awareness, lack of training, lack of management commitment, and low compliance of heat prevention policies. The high proportion of participants not satisfied with current heat prevention measures indicates there is a need for further development of current heat management strategies in the Australian workplaces.

## Supporting Information

S1 Appendix Ethical approval for this study. (JPG)

S2 Appendix Blank questionnaire used in this survey: Professional hygienist perspectives on extreme heat management in the workplace. (PDF)

S3 Appendix Information sheet for survey participants. (PDF)
