# Supplementary figures and images for: Perceptions of Workplace Heat Exposure and Controls among Occupational Hygienists and Relevant Specialists in Australia
Source: PLoS One. 2015 Aug 19;10(8):e0135040. doi: 10.1371/journal.pone.0135040 (PMC4546008; doi:10.1371/journal.pone.0135040)

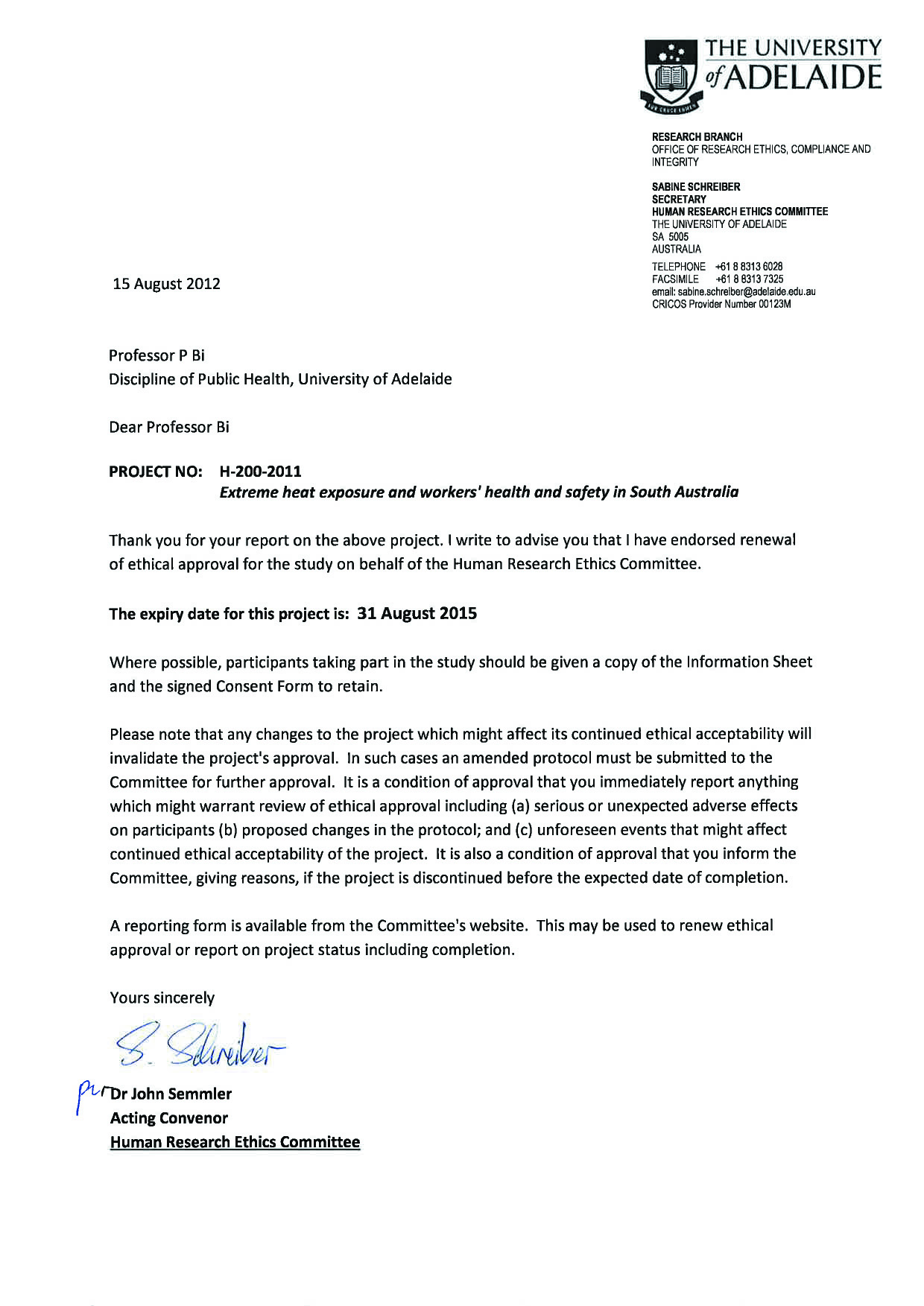

Supplement: S1 Appendix — (JPG) [file pone.0135040.s001.jpg]
