# Supplementary material for: Perceptions of Workplace Heat Exposure and Controls among Occupational Hygienists and Relevant Specialists in Australia
Source: PLoS One. 2015 Aug 19;10(8):e0135040. doi: 10.1371/journal.pone.0135040 (PMC4546008; doi:10.1371/journal.pone.0135040)

THE UNIVERSITY OF ADELAIDE AUSTRALIA

Take 5 minutes to win a $100 prize and $200 to charity

Professional Hygienist Perspectives on Extreme Heat Management in the Workplace

Note: This survey, which is being conducted by The University of Adelaide, is investigating workplace heat exposure and health & safety. Please answer each question by placing a (✓) in the box next to your preferred answer. Return the completed questionnaire to the box on the reception desk, and you will be in the draw to win a $100 Coles-Myer gift voucher and a $200 Christmas donation to an AIOH-sponsored charity. This survey is anonymous to maintain confidentiality. Your contribution to this survey is very greatly appreciated.

Part A: Your circumstances

1. Job Position:

- ☐ Consultant
- ☐ General Industry Hygienist
- ☐ Mining Hygienist
- ☐ Government Hygienist
- ☐ University Hygienist
- ☐ Military Hygienist
- ☐ Health and Safety Manager
- ☐ Other ______________

2. Years of OH&S experience: __________

3. Which state are you from?

- ☐ ACT
- ☐ NSW
- ☐ NT
- ☐ QLD
- ☐ SA
- ☐ TAS
- ☐ VIC
- ☐ WA

Given predictions of increased hot weather and the likelihood of more frequent extreme heat events in Australia we would like to ascertain your thoughts on heat stress in the workplace

Part B: Your perspectives

4. On a scale of 1-5 with 5 being the most concerned, how concerned are you about extreme heat resulting in increased hazards in the workplace?

1	2	3	4	5
☐	☐	☐	☐	☐

5. **Do you agree or disagree that extremely hot weather due to changing climate presents a future challenge for workplace heat management?**

☐ Strongly agree ☐ Agree ☐ Neither agree nor disagree ☐ Disagree ☐ Strongly disagree

6. **Do you know of any organizations planning for increased frequency of extremely hot weather events?**

☐ Yes ☐ No

7. **In your experience have workers ever expressed concern about heat in your workplace (or workplaces you consult in) during very hot weather?**

☐ Yes ☐ No

If yes, how often?

☐ Seldom ☐ Sometimes ☐ Often ☐ Always

8. **In the last five years, have you had to investigate the circumstances around injuries or illnesses that could be attributed to extreme heat (air temperature greater than 38℃)?**

☐ Yes ☐ No

If yes, how many times?

☐ Once ☐ Two to five times ☐ More than five times ☐ Not sure

9. **What measures are adopted in the workplace or workplaces that you consult in during very hot weather?**

(Select all that apply)

☐ Provision of cool drinking water

☐ Broad brimmed hats supplied

☐ Stop outdoor work if the temperature is extreme

☐ Rescheduling work time

☐ Other (specify)______________________

☐ Electric fan

☐ Shady rest area

☐ Central cooling system or air conditioning

☐ Heat stress related training

10. Overall, are you satisfied or dissatisfied with the measures currently adopted for reducing the risk of heat illnesses and injuries in the workplace or workplaces that you consult in during very hot weather?

☐ Strongly satisfied ☐ Satisfied ☐ Neither satisfied nor dissatisfied ☐ Dissatisfied ☐ Strongly dissatisfied

11. Do you think there is a need for more training about working in very hot weather in your workplace or workplaces that you consult in?

☐ Yes → Which aspects should be strengthened? ______________________________

____

☐ No → Because? (Select all that apply)

- ▶ Training is generally adequate......... ☐
- ▶ I don't think it is a serious problem.... ☐
- ▶ Other(specify)________________ ☐

☐ Not sure

12. Is there a hot weather plan or heat stress policy in your workplace or any workplaces that you consult in?

☐ Yes → which industrial sectors? ______________________________

☐ No

☐ Not sure

13. Do you think there should be more guidelines or regulations for reducing the risk of heat-related illnesses and injuries in very hot weather applicable to your workplace or workplaces that you consult in?

☐ Yes

☐ No → Because? (Select all that apply)

- ▶ There are enough regulations......... ☐
- ▶ I don't think it is a serious problem... ☐
- ▶ I haven't thought about it................ ☐
- ▶ Other ________________ ☐

☐ Don't know

14. Do you know of companies that have recently made changes to maintain OHS in work environments where extreme heat may become more common?

☐ Yes ☐ No

If yes, what changes have been made? ______________________________

____

____

15. Do you intend to alter your recommendations to management or companies due to the likelihood of increased hot weather?

☐ Yes

☐ No → Because? (Select all that apply)

☐ Not sure

- ▶ I don't think it is a serious problem....... ☐
- ▶ The timescale is too long.................. ☐
- ▶ I haven't thought about it.................. ☐
- ▶ Other ______________________ ☐

16. What do you foresee as potential barriers for the prevention of heat stress in workplaces?

(Select all that apply)

☐ Lack of awareness

☐ Lack of training

☐ Lack of management commitment

☐ Lack of financial resources to bring in engineering controls

☐ Lack of specific heat-related guidelines and regulations

☐ Low compliance and implementation of heat stress prevention programs

☐ None

☐ Other ____

17. Do you have any further recommendations or suggestions for the prevention of heat-related illnesses and injuries in Australian workplaces?

____

____

____

____

Thank you very much for taking time to complete the questionnaire.

Please return the completed questionnaire to the box on the reception desk by 3:00 pm on Tuesday (4th December), you will be in the draw to win a $100 prize and $200 for an AIOH sponsored charity.

If you have any queries or interest in this research, please contact me at jianjun.xiang@adelaide.edu.au

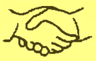

Supplement: S2 Appendix — (PDF) [file pone.0135040.s002.pdf]
