# Supplementary material for: Perceptions of Workplace Heat Exposure and Controls among Occupational Hygienists and Relevant Specialists in Australia
Source: PLoS One. 2015 Aug 19;10(8):e0135040. doi: 10.1371/journal.pone.0135040 (PMC4546008; doi:10.1371/journal.pone.0135040)

AIOH ADELAIDE 2012
MEETING GLOBAL CHALLENGES

AUSTRALIAN INSTITUTE OF OCCUPATIONAL HYGIENISTS INC
30th ANNUAL CONFERENCE & EXHIBITION
ADELAIDE CONVENTION CENTRE | SOUTH AUSTRALIA
1st - 5th DECEMBER 2012

THE UNIVERSITY OF ADELAIDE AUSTRALIA

INFORMATION SHEET

Professional Hygienist Perspectives on Extreme Heat Management in the Workplace

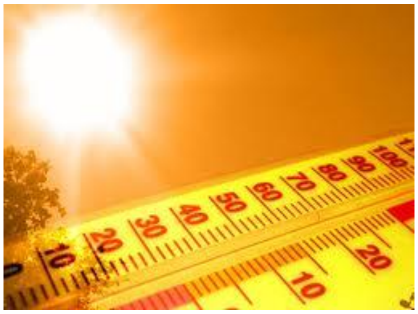

Global warming is likely to lead to more extreme climatic events. The potential impact of climate change has received international attention, and there is a growing body of occupational and environmental hygiene literature on the topic. However, there is a gap in knowledge on the current perspectives and opinions of professional hygienists who provide advice, and particularly in a country where extreme heat episodes are likely to be more common.

Please take 5 minutes to complete the anonymous questionnaire that covers current and future concerns and return in the box at the registration desk by **3:00 pm on Tuesday** (4th December) to be in the draw to **win a $100 Coles-Myer Christmas gift voucher and a $200 Christmas donation to an AIOH-sponsored charity**. The findings of the survey will be provided in a forthcoming AIOH Newsletter.

Ethics approval: University of Adelaide (H-200-2011)

Eligibility: AIOH members attending the AIOH 2012 conference

Your participation is greatly appreciated.

For further information, please contact: Jianjun Xiang, Discipline of Public Health, School of Population Health, the University of Adelaide, jianjun.xiang@adelaide.edu.au

Thanks for your contribution to this survey

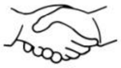

Supplement: S3 Appendix — (PDF) [file pone.0135040.s003.pdf]
